# LW-YOLO11: A Lightweight Arbitrary-Oriented Ship Detection Method Based on Improved YOLO11

**DOI:** 10.3390/s25010065

**Published:** 2024-12-26

**Authors:** Jianwei Huang, Kangbo Wang, Yue Hou, Jiahe Wang

**Affiliations:** 1 College of Power Engineering, Naval University of Engineering, Wuhan 430033, China; 15559110766@163.com (J.H.); houyue1982@163.com (Y.H.); wjhsci7099@163.com (J.W.); 2 Maritime College, Fujian Chuanzheng Communications College, Fuzhou 350007, China; 3 Simulation Training Center, Naval University of Engineering, Wuhan 430033, China

**Keywords:** arbitrary-oriented ship detection, lightweight networks, improved YOLO11, GSConv module, multi-scale dilated attention, cross-stage partial stage

## Abstract

Arbitrary-oriented ship detection has become challenging due to problems of high resolution, poor imaging clarity, and large size differences between targets in remote sensing images. Most of the existing ship detection methods are difficult to use simultaneously to meet the requirements of high accuracy and speed. Therefore, we designed a lightweight and efficient multi-scale feature dilated neck module in the YOLO11 network to achieve the high-precision detection of arbitrary-oriented ships in remote sensing images. Firstly, multi-scale dilated attention is utilized to effectively capture the multi-scale semantic details of ships in remote sensing images. Secondly, the interaction between the spatial information of remote sensing images and the semantic information of low-resolution features of ships is realized by using the cross-stage partial stage. Finally, the GSConv module is introduced to minimize the loss of semantic information on ship features during transmission. The experimental results show that the proposed method has the advantages of light structure and high accuracy, and the ship detection performance is better than the state-of-the-art detection methods. Compared with YOLO11n, it improves 3.1% of mAP@0.5 and 3.3% of mAP@0.5:0.95 on the HRSC2016 dataset and 1.9% of mAP@0.5 and 1.3% of mAP@0.5:0.95 on the MMShip dataset.

## 1. Introduction

Target detection in optical remote sensing images is one of the basic tasks in the field of aerial and satellite image analysis, which aims to classify and localize the target of interest in the image and effectively extract important information about the target to be tested [1,2,3]. Therefore, the realization of accurately detecting ships in optical remote sensing images is of great practical significance and security value in the fields of fishery, maritime search and rescue, maritime traffic management, and combating maritime smuggling [4,5,6].

Traditional target detection methods primarily rely on researchers to design feature extractors [7,8]. However, with the diversification of image features, the design of handmade features has been gradually complicated, which makes it difficult to further improve the performance of traditional detection methods. In particular, optical remote sensing images are characterized by high resolution and complex features, which make traditional detection methods incapable of meeting the needs of remote sensing image target detection.

In recent years, target detection technology based on deep learning has been rapidly developed [9]. At present, the mainstream target detection algorithms are mainly categorized into two-stage detection and one-stage detection. The two-stage detection method is mainly represented by the region-based convolutional neural network (CNN) series of algorithms. The detection steps of the two-stage method are as follows: (1) Firstly, according to a pre-designed algorithm, a large number of region proposals are generated to initially complete the classification of foreground and background. (2) Secondly, the region proposals are filtered by using the detection head to further categorize the target. (3) Finally, the region’s proposal is returned, and the bounding box is fine-tuned. Typical two-stage detection algorithms include R-CNN [10], Fast R-CNN [11], Faster R-CNN [12], SPP-NET [13], and so on. Instead of generating a large number of region proposals, the one-stage detection method extracts features, predicts categories, and localizes targets directly in the network [14,15,16,17]. Therefore, the two-stage method has higher detection accuracy but lower speed. Although the detection accuracy of the one-stage method is usually lower, it has a faster detection speed and satisfies the real-time detection requirements in specific scenarios more effectively.

The target detection algorithms based on deep learning have high accuracy, but due to the numerous model parameters and expensive computational and storage costs, a large amount of computer memory is consumed in initializing the model. Moreover, high-performance graphics cards are required for acceleration to take full advantage. As a result, it is difficult to deploy on platforms with fixed performance and limited computational resources, such as satellites, drones, and other embedded devices for real-world application terminals [18,19,20]. To address this problem, many scholars have carried out research on lightweight remote sensing image ship detection algorithms based on deep learning. Tian et al. constructed a new ship remote sensing image dataset and proposed a lightweight and high-performance ship detection network, which effectively reduces the model parameters and computational complexity [21]. In order to balance the model detection performance and computational speed, Yin et al. proposed a new lightweight framework, called HSI-Ship Detection Net, suitable for deployment in resource-limited platforms, which achieved excellent detection accuracy in small-size ship detection in remote sensing images [22]. Han et al. optimized the YOLOv8n model by designing the SSC2F module and the MC2F structure and constructed a lightweight network SSMA-YOLO to solve the leakage and misdetection problems of ship detection in optical remote sensing images [23]. Although the above optical remote sensing image lightweight ship detection method has the advantages of high precision and fast efficiency and can be deployed on edge devices, it is easy to introduce background pixels and interference factors in the detection of ships in different directions. Particularly, for adjacent ship targets, the detection method based on horizontal boxes suffers from the problem of overlapping bounding boxes, which seriously affects the visualization effect of ship detection and negatively influences the subsequent data analysis work.

Compared with conventional target detection, the arbitrary-oriented target detection algorithm generates more effective rotating bounding boxes [24,25,26]. It solves the problem of overlapping horizontal bounding boxes due to the proximity of the ships, which applies to the scenes with the random distribution of the ship directions in the remote sensing images. Hua et al. designed an end-to-end anchor-free oriented ship detector framework based on a multi-scale dense-point rotated Gaussian heat map, which solves the problems of complex background, interference factors, and size differences in remote sensing images [27]. Ge et al. introduced shape descriptors into remote sensing image processing and proposed an anchor-free detection method based on key points, KeyShip, which realizes the high-precision detection of ships with different angles in remote sensing images [28]. Su et al. improved the YOLO algorithm by designing a novel feature extraction network, DCNDarknet25, to form a fully convolutional lightweight network for ship detection in any direction in large-area remote sensing images, which demonstrated excellent detection performance [29]. Liang et al. proposed a novel symmetric deformable convolutional detector called MidNet using an anchor-free and angle-free paradigm, which reduces the sensitivity of hyperparameter settings to the accuracy of angle regression by encoding each oriented object with one centroid and four centers [30]. The above methods have achieved outstanding results in detecting ships with arbitrary directions in remote sensing images, but still suffer from deficiencies in detection speed, which hardly meets the real-time requirements of remote sensing scenes.

This paper proposes a lightweight arbitrary-oriented ship detection method for remote sensing images based on improved YOLO11, called LW-YOLO, to address the problem that most current optical remote sensing image ship detection methods are not feasible to use to effectively balance detection accuracy and efficiency. Specifically, inspired by the idea of GFPN, a lightweight and effective neck network is designed by introducing a multi-scale dilated attention (MSDA) [31] module and the cross-stage partial stage (CSPStage) [32]. The CSPStage increases the cross-scale connection between neighboring layer features and the cross-layer connection under the same scale features, which makes the information of deep and shallow features and large-scale and small-scale features of the network fully exchanged, to solve the problem of the scarcity of ship feature information in remote sensing images. Furthermore, the YOLO11n [33] network is used as the basic framework of the algorithm, and the lightweight convolution technique, GSConv [34], is introduced to minimize the model parameters and reduce the information loss of ship features during transmission. The contributions of this paper are summarized as follows:

- A novel lightweight neck network is designed to enhance the information interaction between features with different scales by jumping across layers of connections. Moreover, the dependency between sparsity features and distant pixels is constructed to improve the performance of the model in the task of detecting ships with different sizes in remote sensing images.
- To address the problem of information loss in optical remote sensing images during feature space compression and channel expansion, the lightweight convolution technique, GSConv, is introduced to maximize the retention of hidden dependencies between each channel, which maintains the model accuracy and reduces the computational complexity.
- In this paper, ablation experiments and performance comparison tests are conducted to fully demonstrate the effectiveness of the proposed model for ship detection in remote sensing images. The proposed method achieves the best performance of ship detection, achieving 97.6% of mAP@0.5 and 90.1% of mAP@0.5:0.95 on the HRSC2016 dataset and 90.1% of mAP@0.5 and 86.7% of mAP@0.5:0.95 on the MMShip dataset. The model size is only 7.4 M, which can be effectively deployed in a resource-efficient platform.

The remainder of this paper is organized as follows: Section 2 introduces the original model YOLO11 proposed in this paper and the innovations. Section 3 describes the experimental preparations and analyzes the experimental results to demonstrate the ship detection performance and speed of the proposed method. Finally, Section 4 presents the main conclusions and directions for future work.

## 2. Principles and Innovations

### 2.1. YOLO11 Model

**Figure 1:**
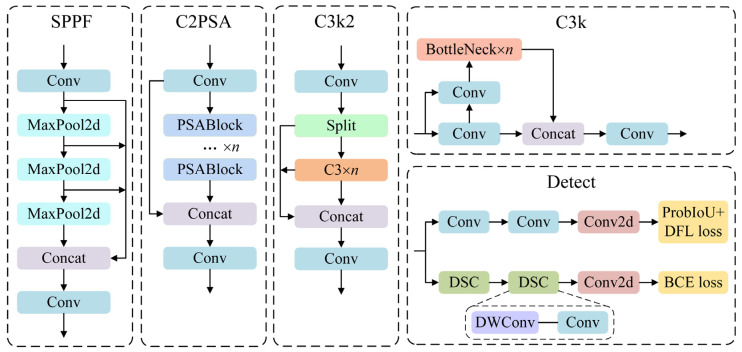
The optimized module structure of YOLO11 network.

The latest version of the Ultralytics YOLO series of real-time target detectors, YOLO11, offers the most excellent detection accuracy, speed, and efficiency. Compared with YOLOv8, YOLO11 replaces the original C2F module with the C3K2 module in the backbone and neck structures. Meanwhile, a C2PSA module, which is similar to the attention mechanism, is added behind the SPPF module to further enhance the ability to extract features from images. Moreover, by introducing the head idea of YOLOv10, YOLO11 uses an anchor-free based decoupled head, in which the regression branch uses normal convolutional blocks and the classification head uses depth-wise separable convolution (DWConv), effectively reducing redundant computation and improving efficiency. The optimized modules of YOLO11 are shown in Figure 1. Overall, the number of model parameters of YOLO11 is reduced by 20%.

### 2.2. Proposed Method

**Figure 2:**
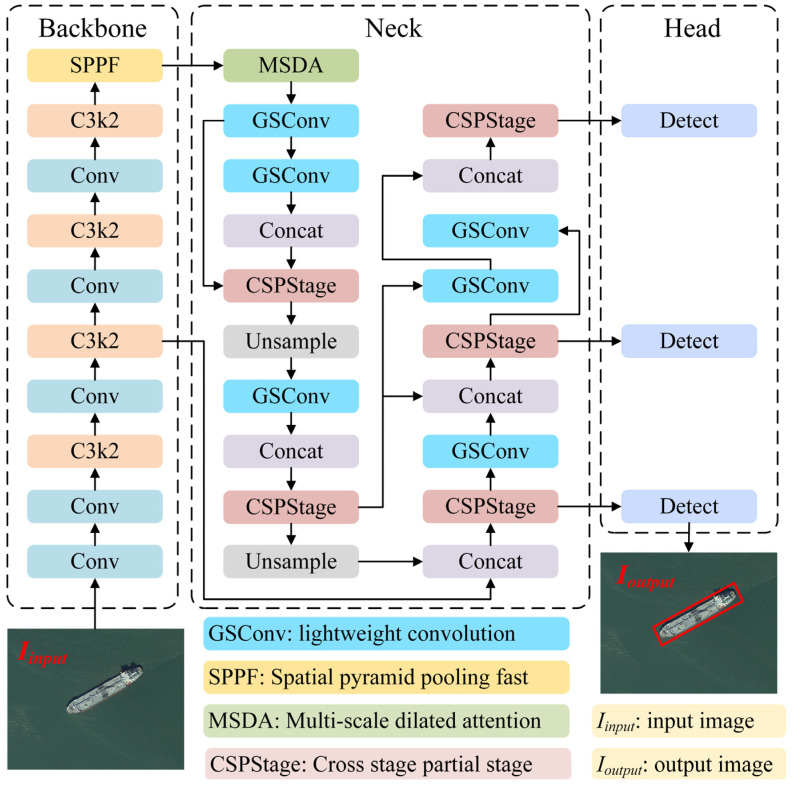
The network structure of the proposed method.

In this paper, an efficient and lightweight neck network is designed to achieve the high-precision and fast detection of ships at any angle in optical remote sensing images. Firstly, the initial feature map is generated from the remote sensing image through the feature extraction module of the backbone network. Secondly, the MSDA module performs long-distance pixel-dependent construction on the feature map output by the SPPF, enriching the context semantic information of the multi-scale feature map. Thirdly, the CSPStage module further captures the underlying semantic information and spatial information of the feature map and realizes the efficient fusion of multi-scale features. Fourthly, the GSConv module is utilized to perform channel compression at the neck to reduce the number of feature map channels and decrease the computational burden while maintaining the integrity of the feature information. In addition, an effective connectivity strategy (such as skip connectivity and residual connectivity) is introduced at appropriate locations in the neck network to ensure effective gradient transfer and efficient feature transfer, thus improving the model’s ability to detect small targets with low resolution. Finally, the processed feature maps are transmitted to the detect module for target classification and regression, completing the detection of arbitrary-oriented ships. The network structure of the proposed method is shown in Figure 2.

#### 2.2.1. MSDA

**Figure 3:**
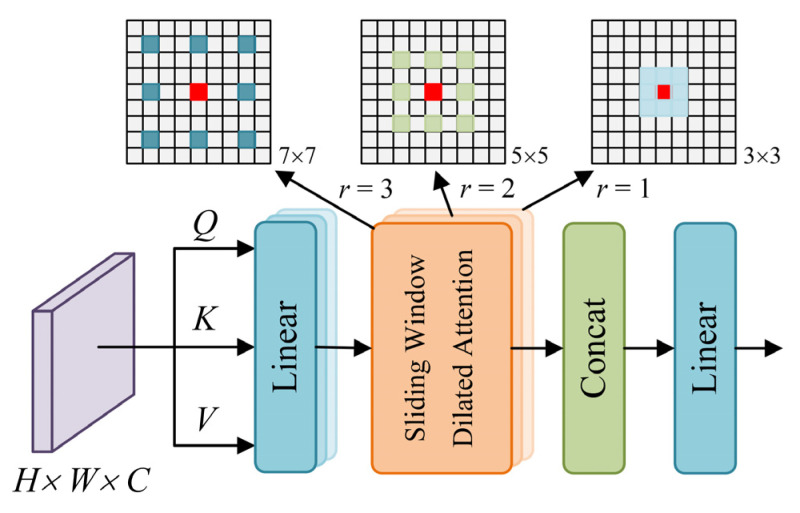
The structure of the MSDA module.

Due to the small scale and inconspicuous features of ships in optical remote sensing images, conventional convolution neural networks can only construct local dependencies of features, ignoring the influence of long-distance pixel dependencies on ship detection. Although vision transformers can use the global attention mechanism to establish a long-distance context dependence of ship features between image blocks, it takes twice the computational cost. Dilated convolution enables the convolution kernel to cover a larger area during sliding by introducing a dilation rate. However, when the dilation rate exceeds 1, the convolution kernel sampling interval increases, resulting in grid-like artifacts in the feature map. Therefore, in order to balance the computational complexity and feature dependency, this paper introduces an MSDA module in the feature fusion network. The MSDA module efficiently captures the sparsity of features at different scales through the self-attention mechanism. The channels of the feature graph are divided into several different heads and perform multi-scale sliding window dilated attention (SWDA) with varying dilation rates to simulate localized and sparse block interactions, thus aggregating multi-scale semantic information and effectively solving the problem of the gridding effect. The structure of the MSDA module is shown in Figure 3.

The MSDA module is designed with multiple heads. For a given feature map $X\in R^{H\times W\times C}$, the corresponding query *Q*, key *K*, and value *V* are obtained by linear projection. The channels of the feature map are divided into *n* different heads and multi-scale SWDA is performed at different heads using different null rates. The process can be described as follows:

(1) $$h_{i}=\mathrm{SWDA}\left(Q_{i},K_{i},V_{i},r_{i}\right),1\leq i\leq n$$

(2) $$X=\mathrm{Linear}\left(\mathrm{Concat}\left[h_{1},\ldots ,h_{n}\right]\right)$$

where *h*_i_ represents the SWDA module output of the *i*-th head, and *r_i_* represents the dilation rate of the *i*-th head.

The MSDA module utilizes the properties of locality and sparsity of the self-attention mechanism to model the long-distance pixel dependency between different scale features, thus eliminating redundant query block information and enriching the multi-scale feature semantic information of the ship. The addition of the MSDA module not only improves computational efficiency but also expands the range of the receptive field, allowing the model to capture complex features more effectively. In addition, the MSDA module can suppress background interference, adaptively aggregate multi-scale features, enhance the perception of small-size and arbitrary-oriented ships, reduce leakage and false detection, increase detection accuracy and robustness, and significantly improve the detection accuracy of ships in remote sensing images.

#### 2.2.2. CSPStage

**Figure 4:**
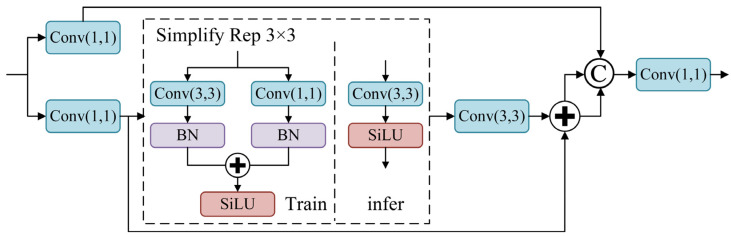
The structure of the CSPStage module.

The idea of RepGFPN is introduced to address the problems of the low utilization of ship features and insufficient fusion expression ability in optical remote sensing images. The C3k2 module in the neck of the YOLO11 algorithm is replaced with the CSPStage feature fusion module to achieve the efficient fusion of multi-scale features. This improves feature utilization efficiency and reduces the computational resource demand of the model. The CSPStage module uses a Conv block to reduce the dimension of the feature channel. Then, the network structure of the reparameterization mechanism is built through the Simplify Rep blocks, where the simplified Rep structure uses a convolution with a kernel of 3 × 3 for training and a convolution with a kernel of 1 × 1 for inference. The structure of the CSPStage module is shown in Figure 4.

For the input features with different scales, the CSPStage module first combines the multi-scale feature information and divides it into upper and lower layers for processing. The upper layer uses only a convolution block with a kernel of 1 × 1. In contrast, the lower layer uses the Simplify Rep module and a convolution block with a kernel of 3 × 3, which completes the staged feature fusion through data transfer to maintain the fluidity of multi-scale feature information. Finally, the feature information of the upper and lower layers is merged by the concatenation operation, and the feature fusion result is output by a Conv block.

#### 2.2.3. GSConv

**Figure 5:**
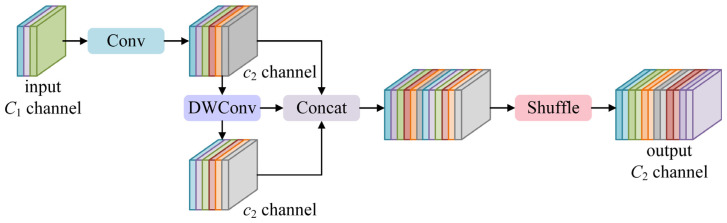
The structure of the GSConv module.

The task of ship detection in remote sensing images often requires real-time performance, which poses a challenge for achieving the light weight of the detection network. However, the Conv block in the neck of the YOLO11 network loses some semantic information when performing feature transfer and fusion. In addition, the Conv block introduces too many parameters during the training process, which makes the ship detection algorithm difficult to deploy on mobile platforms with limited resources. Therefore, a lightweight convolution module GSConv is introduced in the neck, whose structure is shown in Figure 5.

The GSConv module effectively improves the performance of target detection by introducing the ideas of group sparse convolution and group dense convolution. Compared with the traditional convolution block, the GSConv module retains detailed information more effectively when dealing with large-scale targets, thereby improving the accuracy of target detection. Specifically, for features with channel *C*_1_, the GSCONV module first performs a regular convolution operation to generate features with channel *C*_2_/2. Subsequently, a DWConv operation is performed on the feature with channel *C*_2_/2 to obtain a new feature with channel *C*_2_/2 using channel-by-channel convolution and point-by-point convolution techniques. The new feature has the advantages of a small number of parameters and high computational efficiency compared to the original feature, but it contains less feature information. Therefore, it is necessary to perform a concatenation operation on two features with channel *C*_2_/2 to obtain a feature with channel *C*_2_, to compensate for the feature information lost during spatial compression and channel expansion. Finally, the shuffle operation is performed on the features with channel *C*_2_. Through uniform mixing, the feature information from the Conv block and the DWConv block is fully mixed, the hidden dependency between each channel is preserved to the maximum extent, and the local feature information interaction is realized.

## 3. Experiments and Discussion

### 3.1. Environment and Configuration

The experimental environment configuration of this paper uses the Windows 11 operating system with a 13th Gen Intel(R) Core(TM) i9-13900K 3.00 GHz CPU (Intel Corporation, Santa Clara, CA, USA), a RAM of 128 GB, and two GPUs of NVIDIA GeForce RTX 3090 (NVIDIA, Santa Clara, CA, USA). The deep learning framework is Pytorch 2.5.1, the CUDA version is 12.1, and the programming language is Python. The improved YOLO11 network initializes without using the weights of the pre-trained model. Model training completes when the loss function is smooth. The specific hyperparameter information of the model used for training is shown in Table 1.

### 3.2. Dataset

The detection performance of the proposed algorithm is verified on the public remote sensing image ship dataset, HRSC2016, and MMShip. The HRSC2016 dataset contains 2976 ship targets with arbitrary angles and 1061 nearshore background images and sea surface background images, most of which are nearshore background images. Ship samples in remote sensing images are labeled using rotated frames. The image resolution is approximately 1150 × 780 pixels, and the storage format is bmp. The dataset has a complex and diverse image background (including harbors, sea surface, small islands, thin clouds, etc.), a large variation in ship scales, and a dense arrangement of ships in the harbor terminal, which is close to the real application scenarios. The MMShip dataset possesses 5513 near-infrared remote sensing images, including 5016 small-size images of 512 × 512 pixels and 497 large-size images of 10,980 × 10,980 pixels, with the small-size images obtained by cropping from the large-size images. The MMShip dataset has a large amount of data on ship instances, covers the global sea area, and includes a wide range of scenarios involving target occlusion, blurring and texture complexity, and other image characteristics favorable for evaluating ship detection and recognition algorithms. Therefore, the performance test of ship detection using the HRSC2016 dataset and MMShip dataset can effectively verify the practicability of the proposed method in ship detection tasks at sea.

In data preprocessing, unlabeled or incompletely labeled images were removed to ensure the quality of the experimental data. All the categories in the dataset were uniformly changed to a single category: ship. At the same time, the original bounding-box-oriented labeling data in the HRSC2016 dataset were converted to YOLO format, and 1061 ship remote sensing images with complete labeling information were obtained. The original labeling information in the MMShip dataset was horizontal box labeling, which did not contain angular information. Therefore, the X-AnyLabeling labeling tool was used to complete the angular labeling of ship images in the data preparation work. In the experiment, the training set, validation set, and test set were randomly assigned in the ratio of 8:1:1. During the training process, mosaic processing was applied to all training images, and the left–right flip probability of each image was set to 50%.

### 3.3. Performance Evaluation

To objectively evaluate the detection performance of the proposed model, the precision, recall, mAP50, model parameters, model size, and FLOP are introduced as evaluation metrics in this paper [35].

The precision denotes the proportion of the number of positive ship samples predicted by the model to the number of all samples predicted. The precision is calculated as follows:

(3) $$Precision=\frac{TP}{TP+FP}$$

where *TP* denotes a sample that is predicted as ship-positive and is actually also ship-positive, and *FP* denotes a sample that is predicted as ship-positive but is actually ship-negative.

The recall indicates the proportion of the number of ship-positive samples correctly predicted by the model to the number of all positive samples in the dataset. The recall is calculated as follows:

(4) $$Recall=\frac{TP}{TP+FN}$$

where *FN* denotes a sample that is predicted as ship-negative but is actually ship-positive.

The average precision (AP) indicates the area formed by the P-R curve with recall as the *x*-axis and precision as the *y*-axis, and it is calculated as follows:

(5) $$AP=\int_{0}^{1}Precision(Recall)d(Recall)$$

The mean average precision (mAP) is the result obtained from a weighted average of the AP values for all sample categories and is used to measure the detection performance of the model for all ship categories. The mAP is calculated as follows:

(6) $$mAP=\frac{1}{N}\sum_{i=1}^{N}AP_{i}$$

where *AP_i_* denotes the *AP* value of the *i*-th category, and *N* denotes the number of categories in the training dataset. The mAP@0.5 denotes the average accuracy when the IoU of the detection model is set to 0.5. The mAP@0.5:0.95 denotes the average accuracy in the range of IoU values from 0.5 to 0.95.

### 3.4. Ablation Experiments

To validate the enhancement effect of each improvement module on ship detection in optical remote sensing imagery, ablation experiments were conducted on the HRSC2016 dataset, which used the same dataset and experimental setup. The results of the ablation experiments are shown in Table 2.

The introduction of the MSDA modules, mAP@0.5 and mAP@0.5:0.95, improves by about 1%, while the model size, FLOPs, and number of parameters are the same as the original yolo11n model. The effectiveness of the MSDA module in different-scale feature dependency construction and multi-scale semantic information capture is demonstrated. After the introduction of the CSPStage module, the model size increases by 2.3 MB, the FLOPs improve by 2 G, and the number of parameters increases by 1.2 × 10^6^, but the precision and recall are improved, mAP@0.5 improves by 1.3%, and mAP@0.5:0.95 improves by 0.8%. The CSPStage module is demonstrated to improve feature utilization and achieve efficient fusion of multi-scale features. After the introduction of the GSConv module, the FLOPs are reduced by 0.3 G, the model size is reduced by 0.3 MB, the number of parameters is 3.5 × 106, the precision reaches 99.4%, the recall reaches 96.1%, mAP@0.5 reaches 97.6%, and mAP@0.5:0.95 reaches 90.1%. Compared to the YOLO11n method, the proposed method improves by 3.1% on mAP@0.5 and 3.3% on mAP@0.5:0.95, but the model size improves by 1.9 M, FLOPs improve by 1.7 G, and the number of parameters improves by 0.9 × 10^6^. Therefore, the proposed method has the advantages of high precision and light weight, which more effectively meet the requirement of the real-time detection of arbitrary-oriented ships in remote sensing images.

**Figure 6:**
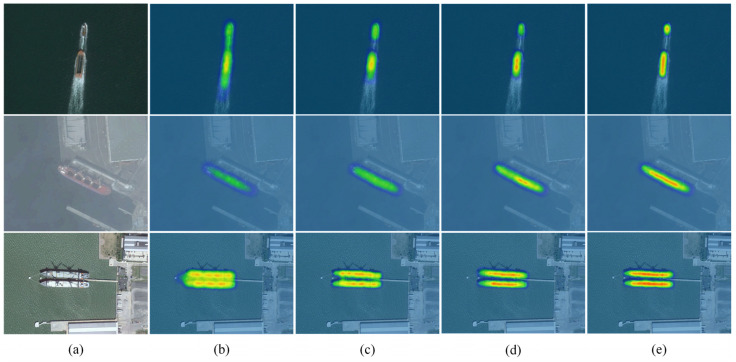
The heat maps generated by different models. (**a**) Input images; (**b**) YOLO11n; (**c**) YOLO11n-MSDA; (**d**) YOLO11N-MSDA-CSPstage; (**e**) LW-YOLO.

To reflect the feature map region that the model focuses on intuitively and easily, a gradient-weighted class activation mapping is introduced for generating the heat maps of YOLO11n and the proposed method. The redder the color of the region in the heat map, the higher the attention of the model. On the contrary, the region with low attention is represented by blue. The heat map results for the three groups of images are shown in Figure 6.

For the image of ships under sail (first row of Figure 6), it is difficult to accurately perceive small-sized ships, although the YOLO11n model can perceive ships roughly. In addition, the yellow area in the thermal image contains waves in addition to the ships involved, and YOLO11n mistakes waves for ship hulls. With the introduction of the MSDA module, CSPStage module, and GSConv module, the yellow area of the heat map gradually narrows down to the ship area, and the color changes to red. As a result, it is demonstrated that the proposed method focuses on the area of small ships with increasing accuracy and attention, effectively suppressing the background interference. For the remote sensing image with blurred texture (second row of Figure 6), the yellow area of the heat map involves the docked ship, but the color is weak and does not cover the overall hull. This shows that the YOLO11n model can only obtain part of the ship’s feature information and cannot capture edge information. While the proposed method pays more and more significant attention to the ship’s features, it can completely extract the ship’s information and effectively differentiate the coast and the hull. Experiments have proven that the improved YOLO11 can detect ships accurately in low-visibility weather, such as fog and rain. This indicates that YOLO11n incorrectly viewed two ships as a single target and that the area of interest included the aisle between the ships. The improved YOLO11 avoids interference problems caused by close distances and focuses attention on the target to be measured. The method enriches the semantic information of multi-scale features by constructing long-range pixel dependencies through the MSDA module, and the introduction of the CSPStage module realizes the effective fusion of multi-scale features and maintains the channel information as much as possible and lightens the network structure under GSConv. Therefore, the proposed method can effectively solve the problem of too close spacing of ships and realize accurate detection and positioning of ships with multiple sizes and arbitrary angles.

### 3.5. Comparison Experiments

#### 3.5.1. Comparison with State-of-the-Art Attention Modules

The attention mechanism is introduced in this paper to guide the model training in the neck feature fusion network, and the effects of multiple attention modules on the performance of ship detection are verified. The experimental results of different attention modules on the HRSC2016 dataset are shown in Table 3.

Compared with YOLO11n, the ship detection of the model is optimized by introducing the GAM module and Biformer module, and mAP@0.5 and mAP@0.5:0.95 are improved by about 0.2% and 0.5%, respectively. However, they require more model-occupied memory and produce a larger number of FLOPs and parameters, making them unsuitable for deployment on resource-limited edge platforms. The EMA module, LSK module, and MSDA module showed similar test results in terms of model size, detection time, and number of parameters, but the MSDA module showed the best ship detection performance, with 0.9% and 0.7% improvement on mAP@0.5 and mAP@0.5:0.95, respectively. Therefore, adding an MSDA module to the neck of the YOLO11 network can not only maintain the light weight of the model but also improve the accuracy of ship detection.

#### 3.5.2. Comparison with State-of-the-Art Detection Method

To further validate the superiority of the proposed algorithm, comparison experiments with the current mainstream OBB detection algorithms are conducted. Gliding Vertex, R^3^Det, Oriented RCNN, RoI-Transformer, H2RBox-v2, PSC, YOLOv8n-OBB, and YOLO11n-OBB algorithms are introduced for the comparison of the detection performance of arbitrary-oriented ships in remote sensing images. The experimental results are shown in Table 4 and Table 5.

On the HRSC2016 dataset, although the Gliding Vertex, R^3^Det, Oriented RCNN, RoI-Transformer, H2RBox-v2, and PSC methods have achieved good mAP@0.5 and mAP@0.5:0.95 in the ship detection of remote sensing images, they have too many model parameters, resulting in excessive storage space. Although the YOLO series networks have low FLOPs, they have the advantages of small model sizes and few parameters. In the case of the YOLOv8n-OBB model with a model size of 7.5 MB and a parameter of 3.1 M, mAP@0.5 reaches 93.7%, and mAP@0.5:0.95 reaches 86.2%. YOLO11n-OBB achieves better performance under the conditions of smaller model size and fewer parameters. Although the proposed method is slightly larger than YOLO11n-OBB in model size and parameter quantity, it achieves the best performance: mAP@0.5 reaches 97.6%, and mAP@0.5:0.95 reaches 90.1%. Compared to YOLO11n-OBB, the proposed method has an enhancement of 3.1% on mAP@0.5 and 3.3% on mAP@0.5:0.95. Therefore, the proposed method achieves the high-precision detection performance of ships in any direction at the cost of smaller storage space and has the advantages of a lightweight structure and rapid detection.

On the MMShip dataset, the training and test images involve target occlusion, blurring, and the influence of interfering factors, which leads to the phenomenon of degradation in the detection effect of all algorithms. The Gliding Vertex method achieves only 83.1% of mAP@0.5 and 81.3% of mAP@0.5:0.95 in the ship detection experiments. The R^3^Det, Oriented RCNN, RoI-Transformer, and H2RBox-v2 methods have better detection performance than the Gliding Vertex, but they are more memory-intensive. The PSC method has superior detection results compared to YOLOv8n-OBB, achieving 87.9% of mAP@0.5 and 85.1% of mAP@0.5:0.95, but it requires more storage memory and parameters. The YOLO11n-OBB method has the smallest model size of 5.55 M and achieves excellent detection accuracies, with 88.2% for mAP@0.5 and 85.4% for mAP@0.5:0.95. Compared to YOLO11n-OBB, the proposed method achieves the highest ship detection scores with 90.1% of mAP@0.5 and 86.7% of mAP@0.5:0.95, despite the 1.9 M boost in model size. In conclusion, the method in this paper can excellently fulfill the remote sensing ship detection tasks in different scenes and effectively deal with the problems of texture blurring, target occlusion, and many interfering factors.

**Figure 7:**
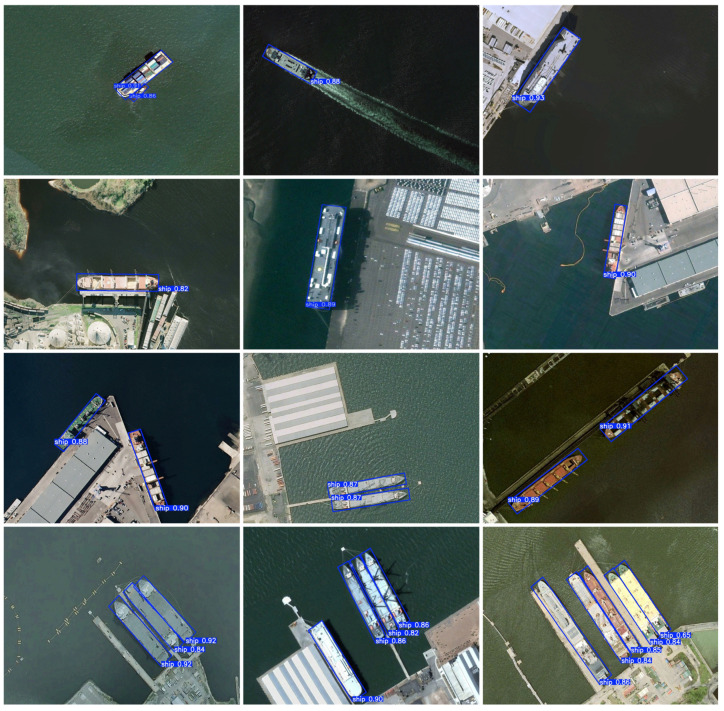
The ship detection results of the proposed method on the HRSC2016 dataset.

**Figure 8:**
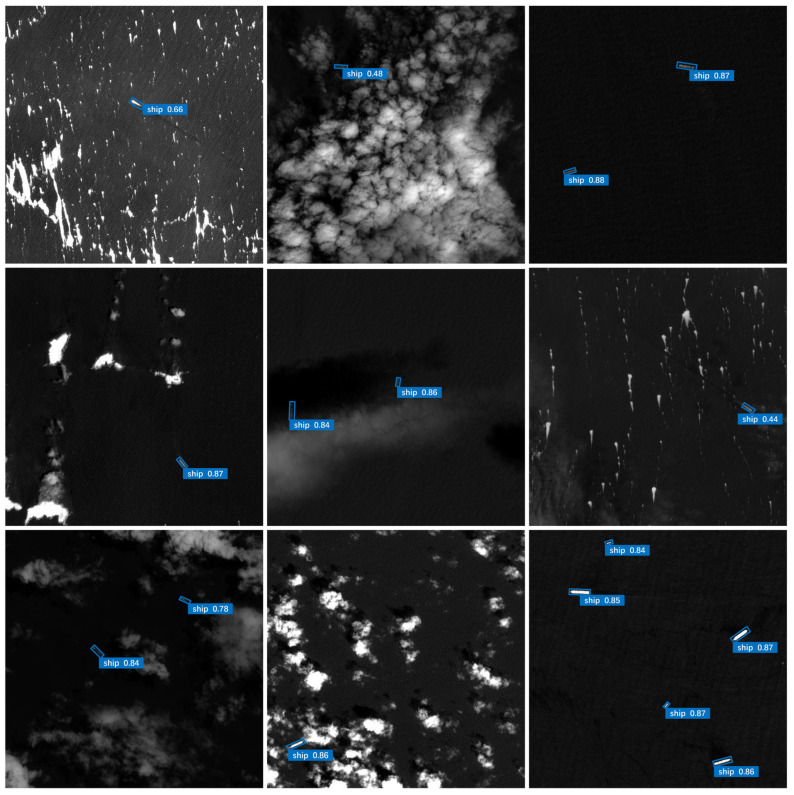
The ship detection results of proposed method on the MMShip dataset.

To perceive the improvement effect of this paper’s algorithm more clearly, a picture-by-picture detection is carried out on the test set, and representative data in complex backgrounds with large scale differences and dense distribution and small differentiation from the background are selected for visualization experiments. The ship detection results are shown in Figure 7 and Figure 8. For single-target images, the proposed method accomplishes the detection task with a rotating bounding box, whether it is a ship traveling at sea or a ship docked at the shore. For multi-target images, the proposed method not only detects spatially located neighboring ships one by one, but also achieves the accurate detection of ships with different sizes, demonstrating the superior detection performance of the model. In near-infrared remote sensing images, the proposed method can accurately discriminate between interfering factors and targets to be detected. For complex background images, the proposed method correctly detects ships. In target occlusion and imaging blurred scenes, the proposed method effectively captures ship feature information and detects small-sized ships.

## 4. Conclusions

The purpose of this study is to improve the real-time detection ability of ships in remote sensing images and to achieve the high-precision detection of ships of different sizes and in any direction under complex backgrounds. Therefore, a lightweight ship detection method in any direction based on improved YOLO11 is proposed to solve the problem that the detection accuracy and efficiency cannot be effectively balanced. A lightweight and efficient feature fusion neck network is proposed to enhance the information interaction between different scale features by skipping across the connection layer. The MSDA module is introduced to construct the dependence between sparse features and remote pixels. The CSPStage module is used to capture multi-scale semantic information and achieve efficient feature fusion. Through the GSConv module, the hidden dependencies between feature channels are retained, and the calculation speed of the model is improved. The experimental results show that compared with the advanced detection methods, the proposed method shows superior ship detection performance, achieving 97.6% mAP@0.5 and 90.1% mAP@0.5:0.95 on the HRSC2016 dataset and achieving 90.1% mAP@0.5 and 86.7% mAP@0.5:0.95 on the MMShip dataset, and the detection speed meets the requirements of real-time ship detection.

In the future, we plan to deploy the proposed lightweight ship detection model on high-performance edge computing devices to further test and optimize the performance of the model.

## Figures and Tables

**Table 1:** The specific hyperparameter information of the model.

Hyperparameters	Value
Learning Rate	0.001
Momentum	0.937
Epochs	2000
Batch Size	24
Images Size	640 × 640
Optimizer	SGD
Weight Decay	0.0005
Patience	100
Work	8
Pretrained	False
Warmup Epochs	3
Warmup Momentum	0.8
Warmup Bias Learning Rate	0.1

**Table 2:** The detection effect of the improvements in the proposed method.

YOLO11n	MSDA	CSPStage	GSConv	mAP@0.5	mAP@0.5:0.95	Model Size/MB	FLOPs/G	Parameter/10^6^
√				94.5%	86.8%	5.5	6.6	2.6
√	√			95.4%	87.5%	5.5	6.6	2.6
√	√	√		96.7%	88.3%	7.8	8.6	3.8
√	√	√	√	97.6%	90.1%	7.4	8.3	3.5

‘’ √ ‘’ denotes the model or module used in the test.

**Table 3:** The performance comparison of different attention modules.

Method	mAP@0.5	mAP@0.5:0.95	Model Size/MB	FLOPs/G	Parameter/10^6^
YOLO11n [33]	94.5%	86.8%	5.5	0.6	2.6
YOLO11n + GAM [36]	94.7%	86.9%	9.7	14.8	4.9
YOLO11n + Biformer [37]	94.9%	87.3%	12.6	17.5	2.9
YOLO11n + EMA [38]	95.0%	87.1%	6.0	1.1	2.6
YOLO11n + LSK [39]	95.3%	87.3%	5.9	1.0	3.0
YOLO11n + MSDA	95.4%	87.5%	5.5	0.7	2.6

**Table 4:** The performance comparison of different detection methods on the HRSC2016 dataset.

Method	mAP@0.5	mAP@0.5:0.95	Model Size/MB	FLOPs/G	Parameter/10^6^
Gliding Vertex [40]	90.2%	84.7%	44.5	121.5	41.1
R^3^Det [41]	91.2%	84.6%	44.7	200.9	41.6
Oriented RCNN [42]	92.3%	86.1%	125.1	41.1	121.6
RoI-Transformer [43]	92.2%	85.9%	125.5	55.1	122.6
H2RBox-v2 [44]	91.7%	85.3%	54.2	242.7	51.4
PSC [45]	92.0%	85.8%	39.4	210.9	36.2
YOLOv8n-OBB [24]	93.7%	86.2%	7.5	8.3	3.1
YOLO11n-OBB [33]	94.5%	86.8%	5.5	6.6	2.6
Ours	97.6%	90.1%	7.4	8.3	3.5

**Table 5:** The performance comparison of different detection methods on the MMship dataset.

Method	mAP@0.5	mAP@0.5:0.95	Model Size/MB	FLOPs/G	Parameter/10^6^
Gliding Vertex [40]	83.1%	81.3%	44.5	127.4	45.9
R^3^Det [41]	84.2%	82.5%	44.7	214.6	46.2
Oriented RCNN [42]	84.9%	83.2%	125.1	47.9	127.4
RoI-Transformer [43]	86.7%	83.8%	125.5	59.3	132.2
H2RBox-v2 [44]	87.2%	84.6%	54.2	246.8	55.6
PSC [45]	87.9%	85.1%	39.4	221.4	39.4
YOLOv8n-OBB [24]	86.6%	84.2%	7.5	12.6	4.8
YOLO11n-OBB [33]	88.2%	85.4%	5.5	9.9	4.7
Ours	90.1%	86.7%	7.4	12.4	5.2

## Data Availability

The data presented in this study are available on request from the corresponding author.
